# Highly Stretchable and Rapid Self-Recoverable Cryogels Based on Butyl Rubber as Reusable Sorbent

**DOI:** 10.3390/gels5010001

**Published:** 2019-01-07

**Authors:** Sevil Muslumova, Berkant Yetiskin, Oguz Okay

**Affiliations:** Department of Chemistry, Istanbul Technical University, Maslak, Istanbul 34469, Turkey; muslumova15@itu.edu.tr (S.M.); yetiskinb@itu.edu.tr (B.Y.)

**Keywords:** organogels, cryogels, butyl rubber, macroporous rubber gels, mechanical properties

## Abstract

Cryogels based on hydrophobic polymers combining good mechanical properties with fast responsivity are attractive materials for many applications, such as oil spill removal from water and passive sampler for organic pollutants. We present, here, cryogels based on butyl rubber (BR) with a high stretchability, rapid self-recoverability, and excellent reusability for organic solvents. BR cryogels were prepared at subzero temperatures in cyclohexane and benzene at various BR concentrations in the presence of sulfur monochloride (S_2_Cl_2_) as a crosslinker. Although the properties of BR cryogels are independent of the amount of the crosslinker above a critical value, the type of the solvent, the cryogelation temperature, as well as the rubber content significantly affect their properties. It was found that benzene produces larger pore volumes as compared to cyclohexane due to the phase separation of BR from benzene at low temperatures, producing additional pores. Increasing cryogelation temperature from −18 to −2 °C leads to the formation of more ordered and aligned pores in the cryogels. Increasing BR content decreases the amount of unfrozen microphase of the frozen reaction solution, leading to a decrease in the total porosity of the cryogels and the average diameter of pores. Cryogels formed at −2 °C and at 5% (*w*/*v*) BR in cyclohexane sustain up to around 1400% stretch ratios. Cryogels swollen in toluene can completely be squeezed under strain during which toluene is released from their pores, whereas addition of toluene to the squeezed cryogels leads to recovery of their original shapes.

## 1. Introduction

Polymeric gels combining elasticity and stiffness are viscoelastic smart materials used in a large number of application areas [1]. Among them, organogels, which are hydrophobic polymer networks swollen in organic solvents, have drawn particular attention in the last decade [2]. In contrast to hydrogels that are mainly used in biological and biomedical applications [3,4,5,6,7,8], organogels containing organic solvents are suitable for alternative application areas because of their hydrophobicities. However, both chemically crosslinked hydrogels and organogels typically exhibit poor mechanical properties, and a slow rate of response to the external stimuli [9,10,11]. It was found that an effective energy dissipation mechanism in the gels plays a vital role in their mechanical performances [12]. To improve the mechanical properties, several techniques have been developed, including topological gels [13,14], double-network gels [15,16], nanocomposite gels [17,18], and hydrophobic association gels [19,20]. Moreover, increasing the response rate of gels requires decreasing the gel size to submicrometer range because the response rate is inversely proportional to the square of the gel size [21]. Alternatively, macroscopic sized gels can be made fast-responsive by creating an interconnected pore structure, providing liquid transfer through the pores by convection, which is much faster than diffusion processes. Although various methods have been developed to create fast-responsive macroporous gels, such as phase separation, salt leaching, porogen templating, gas foaming, and electrospinning [22,23,24,25], they are generally brittle, limiting their potential applications.

The challenge for combining good mechanical performance with fast responsivity in a single gel material was achieved successfully by the application of cryogelation technique to the preparation of hydrogels and organogels, as pioneered by Lozinsky [26,27]. Cryogelation is a gelation process conducted under cryotropic conditions, i.e., at a temperature below the freezing temperature of the reaction solution [28,29]. Cryogelation has attracted significant interest in the last decade because it provides formation of mechanically strong, tough, and squeezable gels with fast responsivity due to their interconnected macroporous structure with thick pore walls.

**Scheme 1:**
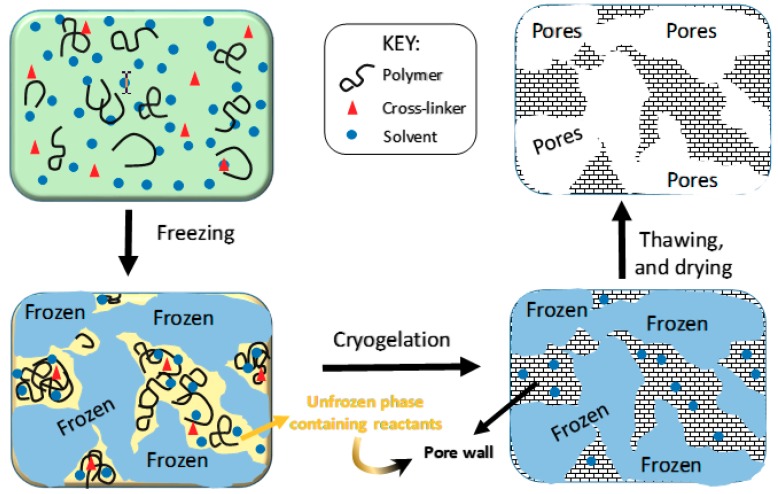
Cartoon showing cryogelation of a polymer solution containing a chemical crosslinker.

In order to obtain cryogels, a polymer or monomer solution containing a crosslinker together with, or without, an initiator, is first frozen at a subzero temperature, i.e., below the crystallization point of solvent. This step leads to the formation of an apparently frozen system consisting of solvent crystals and unfrozen liquid microphase [26] (Scheme 1). Gelation reactions take place in this unfrozen microphase and, after thawing, the macroporous cryogel is obtained due to the in situ-formed template, i.e., solvent crystals. The important point is that, during the freezing step, all the reactants should be concentrated in the unfrozen domains of the apparently frozen system. This phenomenon, called cryoconcentration, allows for the occurrence of the gelation reactions at a high rate, even at very low temperatures and reactant concentrations [28].

Butyl rubber (BR) is a low-cost synthetic copolymer elastomer consisting of isobutylene and isoprene units with a molar ratio in the range of 30/1 to 200/1. Sticky and soft linear BR can be turned into an elastic material by its crosslinking reactions, called vulcanization, discovered by Goodyear in 1839 [30,31]. Vulcanization of rubber with sulfur or sulfur compounds has been a revolutionary method, because it provides fabrication of automobile tires that have radically improved the car and tire industry. It was shown that the vulcanization of several rubbers, including BR, can be conducted in organic solutions in the presence of sulfur monochloride (S_2_Cl_2_) as a crosslinker [32]. The crosslinking reactions using S_2_Cl_2_ can be conducted at above or below the freezing temperature of the organic solvent to produce organogels or organo-cryogels, respectively [32,33,34,35,36].

Owing to the hydrophobicity, squeezability, and fast responsivity, rubber cryogels can be used in various applications, such as sorbents, for the removal of crude oil, petroleum products, and polycyclic aromatic hydrocarbons (PAHs) from aquatic environments [35]. However, the effectivity of rubber cryogels is limited for their use as passive sampler to monitor the concentration of PAHs in the marine environment [37]. This is due to the fact that PAHs have very low solubilities in water and, hence, they exist at a very low concentration, so that their monitoring in aquatic environment requires a passive sampler with a tunable porous structure together with a good mechanical performance. 

The present work aims to optimize the formation conditions of BR cryogels to prepare mechanically strong materials with various pore sizes, size distributions suitable for sorbents, and passive samplers. We prepared BR cryogels at both −2 and −18 °C in the presence of various S_2_Cl_2_ concentrations in two different solvents, namely in benzene and cyclohexane, having freezing temperatures of 5.5 and 6.5 °C, respectively. In our previous work [32,33,34,35,36], the BR content during cryogelation was varied up to 5% (*w*/*v*). As will be seen below, by changing the BR concentration between 5% and 20% (*w*/*v*), cryogels with tunable mechanical properties could be obtained. For instance, the highest fracture stress (614 ± 17 kPa) and stretchability (1400%) so far reported for BR cryogels were achieved by conducting the cryogelation reactions at −2 °C and at 20% and 5% (*w*/*v*) BR, respectively.

## 2. Results and Discussion

Cryogelation reactions were carried out both in benzene and cyclohexane to determine the possible effect of the solvent on cryogel properties. In the following, butyl rubber (BR) cryogels formed in benzene and cyclohexane are named as BR-B and BR-C, respectively. Moreover, BR and S_2_Cl_2_ concentrations are given by BR% (*w*/*v*) (with respect to solvent) and S_2_Cl_2_% (*v*/*w*) (with respect to butyl rubber), respectively. In the first set of experiments, BR concentration was fixed at 5% (*w*/*v*), whereas the crosslinker (S_2_Cl_2_) concentration was varied between 0% and 20% (*v*/*w*). In the second set, BR concentration was changed between 5% and 20% (*w*/*v*) at 10% (*v*/*w*) S_2_Cl_2_. Two different cryogelation temperatures *T*_cry_ were used, namely −2 and −18 °C, which are about 8 and 24 °C below the freezing points of the solvents, respectively.

### 2.1. Swelling Behavior and Porosity of the Cryogels

In order to determine the optimum concentration of the crosslinker S_2_Cl_2_ for the preparation of BR cryogels, experiments were carried out at 5% (*w*/*v*) BR, but at various level of S_2_Cl_2_ between 1 and 20% (*v*/*w*). It was found that the gel fraction *W*_g_ rapidly increases with increasing crosslinker content and reaches unity at 6% (*v*/*w*) S_2_Cl_2_ indicating that all BR molecules are incorporated into the 3D network (Figure S1). A further increase in S_2_Cl_2_ content, up to 20% (*v*/*w*), did not affect the *W*_g_ value, and it remained at around unity. Moreover, the swelling ratio of the cryogels was also independent of the content of the crosslinker S_2_Cl_2_ (Figure S2). In the following, the crosslinker content was fixed at 10% (*v*/*w*) whereas the BR concentration varied between 5% and 20% (*w*/*v*). A complete conversion of BR into the 3D network structure was observed over the whole range of BR% (*w*/*v*) (Figure S3).

**Figure 1:**
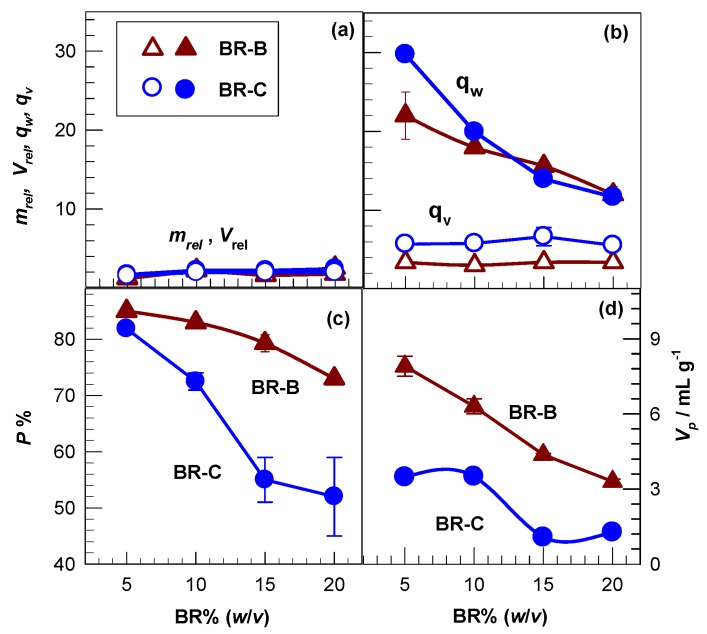
Weight and volume swelling ratios *m*_rel_, *V*_rel_, *q*_w_, and *q*_v_ (**a**,**b**), swollen state porosities *P* (**c**), and pore volumes *V*_p_ (**d**) of BR-B (triangles) and BR-C cryogels (circles) shown as a function of BR% (*w*/*v*). *T*_cry_ = −18 °C. S_2_Cl_2_ = 10% (*v*/*w*). (*n* = 5, M ± SD).

Figure 1a shows relative weight *m*_rel_ and volume swelling ratios *V*_rel_ of the cryogels, calculated with respect to their as-prepared states, plotted against BR% (*w*/*v*). Cryogels formed in benzene (BR-B) and in cyclohexane (BR-C) are shown by triangles and circles, respectively. Both *m*_rel_ and *V*_rel_ do not change much with the rubber content and they remain at 1.5 ± 0.2, independent of the type of cryogelation solvent. This indicates that the mass or volume of the cryogels increases only by 50% when immersed in an excess of toluene, which is a good solvent for BR. However, when the weight (*q*_w_) and volume swelling ratios (*q*_v_) of the cryogels are measured with respect to their dried states, a different behavior appears (Figure 1b). Although the volume swelling ratio *q*_v_ remains at a low level over the whole range of rubber concentration, i.e., at 3.3 ± 0.2 and 5.9 ± 0.5 for BR-B and BR-C cryogels, respectively, weight swelling ratios *q*_w_ are much larger than the *q*_v_ values, and they significantly depend on the rubber concentration. For instance, at 5% (*w*/*v*) BR, *q*_w_ of BR-C cryogel is 6-fold larger than its *q*_v_ value (30 vs. 5) and the difference between *q*_w_ and *q*_v_ decreases as BR% (*w*/*v*) is increased, and they approach each other at 20% (*w*/*v*) BR.

The large difference between *q*_w_ and *q*_v_ at low BR% (*w*/*v*) is attributed to the high porosity of cryogels [22]. Since filling of the pores by a solvent will mainly increase the mass of the cryogel, whereas its volume will not change much, this will lead to a higher swelling ratio with respect to mass as compared to the volume. One may estimate the swollen state porosity (*P*) of the cryogels from their *q*_w_ and *q*_v_ values using the equation [22]

(1) $$P\;\%=\left(1-\frac{q_{v}}{1+(q_{w}-1)d_{2}/d_{1}}\right)\times 100,$$

where *d*_1_ and *d*_2_ are the densities of toluene (0.867 g/mL) and BR (0.92 g/mL), respectively. Figure 1c shows the swollen state porosities *P* of the cryogels plotted against BR% (*w*/*v*). Both BR-B and BR-C have around 84% porosity at 5% (*w*/*v*) BR, whereas it decreases with increasing BR content and becomes 55% and 75% at 20% (*w*/*v*) BR for BR-C and BR-B cryogels, respectively. The results also show that the cryogels formed in benzene (BR-B) have higher porosities as compared to those formed in cyclohexane (BR-C). To verify this finding, pore volume measurements were also carried out as described in the experimental section. Figure 1d shows BR concentration dependence of the pore volume *V*_p_ of BR-C and BR-B cryogels. In accord with the porosity results, *V*_p_ decreases with increasing rubber concentration and benzene creates a larger pore volume as compared to cyclohexane. We attribute this finding to the different extent of interactions between BR and the solvents. Benzene is known to be a poor solvent for BR at low temperatures down to its melting temperature, whereas cyclohexane is a good solvent over all temperatures [36]. Thus, cooling-induced phase separation of BR in benzene solutions during the initial cooling step of cryogelation creates additional pores in the final cryogels, leading to higher porosities in BR-B cryogels as compared to BR-C ones formed in cyclohexane.

### 2.2. Amount of Unfrozen Solvent during Cryogelation

**Figure 2:**
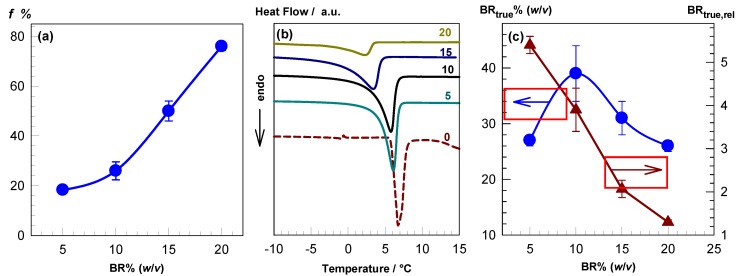
(**a**) Fraction *f* of the unfrozen domains in cyclohexane solution of BR at −18 °C plotted against BR% (*w*/*v*). (*n* = 5, M ± SD). (**b**) DSC thermograms of BR solutions in cyclohexane. BR concentrations (in % (*w*/*v*)) are indicated. (**c**) True BR concentration (BR_true_%) in the unfrozen domains at −18 °C (circles) and its relative value (BR_true,rel_) (triangles) as a function of BR% (*w*/*v*). The arrows indicate the applicable axes.

Measurement of the amount of unfrozen solvent under cryogelation conditions implicitly supported porosity results of the previous section. Figure 2a shows the weight fraction *f* of unfrozen domains in cyclohexane containing 5–20% (*w*/*v*) BR at −18 °C plotted against BR% (*w*/*v*). Increasing the BR content of the solution increases the unfrozen liquid phase fraction *f*, and it approaches to 80% at 20% (*w*/*v*) BR. This reveals that the lower porosities and pore volumes of the cryogels prepared at high rubber contents are due to the decreasing fraction of frozen domains in the reaction system acting as template to form pores. Rubber concentration-dependent variation of the amount of unfrozen domains can be explained with the freezing point depression of cyclohexane caused by butyl rubber. Figure 2b shows the DSC curves of BR solutions at various concentrations. The endothermic peak corresponding to the melting temperature of the solutions shifts to lower temperatures with increasing rubber concentration. For instance, increasing BR concentration from 5% to 20% (*w*/*v*) results in a shift in the freezing point from 6.8 to 2.3 °C, whereas the percentage of the unfrozen domains *f*% increases from about 18% to 80% (Figure 2a). The results thus show significant effect of the rubber content of the reaction system and suggest a transition from macroporous cryogels to non-porous organogels as the rubber concentration is increased.

Cryoconcentration effect, that is, increased BR concentration in the unfrozen regions of the reaction system is presented in Figure 2c, where true BR concentration in the unfrozen domains (BR_true_%) and its normalized value with respect to the nominal value (BR_true,rel_) are plotted against BR% (*w*/*v*). A maximum in BR_true_ of around 40% (*w*/*v*) was measured at 10% (*w*/*v*) nominal BR concentration reflecting drastic increase of rubber concentration in the unfrozen domains during cryogelation. A complete conversion of rubber into the 3D rubber network can thus be explained with this significant cryoconcentration effect dominating more than the decrease of the crosslinking rates due to the lowering temperature. Moreover, normalized value of BR_true_ (BR_true,rel_) is around 5.5 at 5% (*w*/*v*) BR reflecting a 5.5-fold increase of BR concentration in the unfrozen domains. The BR_true,rel_ value continuously decreases with BR content of the solution due to the freezing point depression, as discussed above. 

### 2.3. Morphological Properties of Cryogels

**Figure 3:**
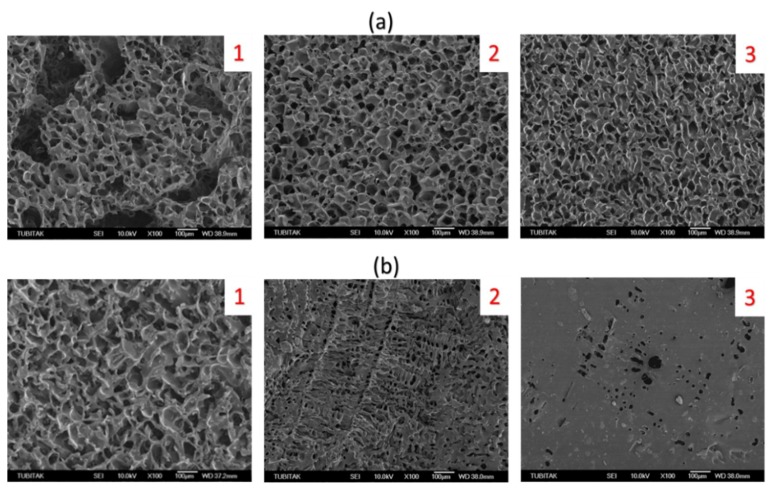
SEM images of BR-B (**a**) and BR-C cryogels (**b**) prepared at 5 (**1**), 15 (**2**), and 20% (*w*/*v*) BR (**3**). S_2_Cl_2_ = 10% (*v*/*w*). *T*_cry_ = −18 °C. Scaling bars are 100 µm.

Scanning electron microscopy (SEM) measurements were conducted on cryogels at various magnifications. Figure 3a,b show SEM images of BR-B and BR-C cryogels, respectively, prepared at −18 °C and at various BR concentrations between 5% and 20% (*w*/*v*). At the lowest BR% (*w*/*v*) (left panel, a1), BR-B cryogels exhibit a wide pore size distribution between 40 and 60 µm, together with pores larger than 100 µm, owing to the phase separation of benzene. The pores in BR-B cryogels become increasingly uniform as BR concentration is decreased. By contrast, BR-C cryogels exhibit relatively uniform pore size distribution, even at the lowest BR% (*w*/*v*), which we attribute to the fact that cyclohexane is a good solvent, even at temperatures close to the freezing point and, hence, a phase separation does not occur during cryogelation [36]. The image 3 in Figure 3b also shows that BR-C cryogels formed at 20% (*w*/*v*) BR are almost non-porous, which is in accord with the results of the previous section. As seen in Figure 2a, the amount of the unfrozen liquid phase *f* drastically increases above 10% (*w*/*v*) BR and becomes around 80% at 20% (*w*/*v*) BR. Thus, the reaction solution containing 20% (*w*/*v*) BR is almost unfrozen at the cryogelation temperature of −18 °C, so that an organogel, instead of a cryogel, forms.

**Figure 4:**
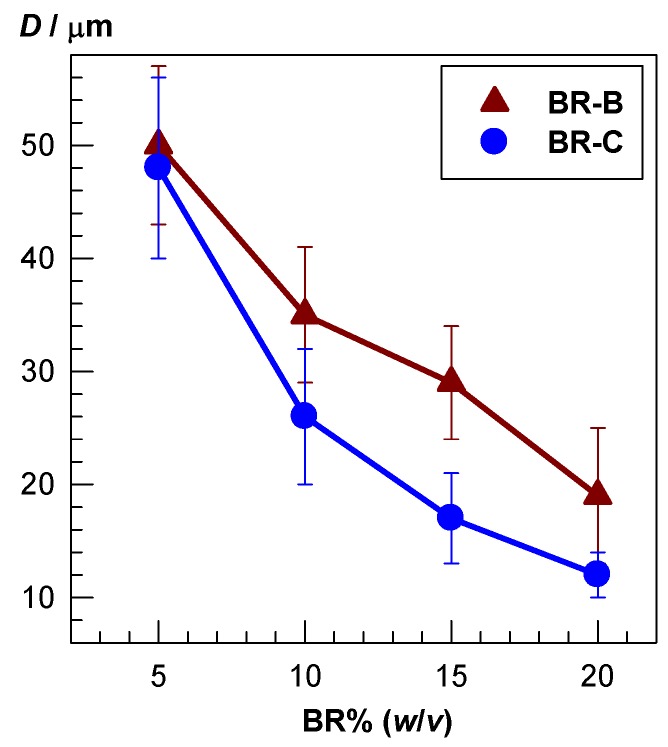
Average pore diameter *D* of BR-B (triangles) and BR-C (circles) cryogels plotted against the BR% (*w*/*v*). *T*_cry_ = −18 °C. (*n* = 5, M ± SD).

Average pore diameters *D* of the cryogels were determined by analyzing their SEM images taken at various magnifications. The results are shown in Figure 4 plotted against BR% (*w*/*v*). At 5% (*w*/*v*) BR, the average pore diameter *D* is 49 ± 8 µm for both BR-B and BR-C cryogels, whereas it decreases with increasing BR content and, at 20% (*w*/*v*) BR, it becomes 19 ± 6 and 12 ± 2 µm for BR-B and BR-C cryogels, respectively. Thus, the pore size of the cryogels can easily be tuned within the micrometer range by changing BR concentration at gelation. The results also show that, above 5% (*w*/*v*) BR, the average pore diameter of BR-B cryogels is larger than that of BR-C ones, due to the phase separation of benzene.

**Figure 5:**
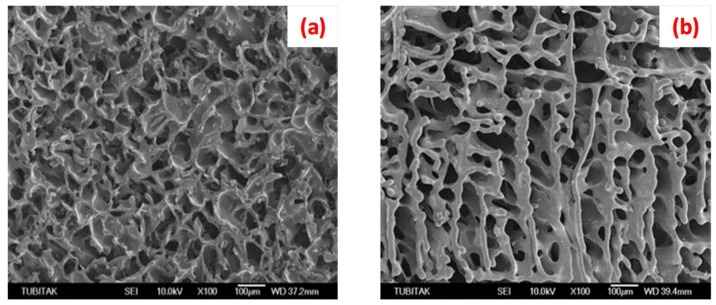
SEM images of BR-C cryogels prepared *T*_cry_ = −18 (**a**) and −2 °C (**b**). BR = 5% (*w*/*v*). S_2_Cl_2_ = 10% (*v*/*w*). Scaling bars are 100 µm.

The effect of the crosslinker (S_2_Cl_2_) amount on the morphologies of the cryogels was also investigated by SEM analysis. Similar to their swelling behavior (Figure S2), the pore morphology of the cryogels was not affected from the amount of S_2_Cl_2_ in the gelation solution (Figures S2 and S4). By contrast, the cryogelation temperature *T*_cry_ significantly affected the morphologies of the cryogels. For instance, Figure 5a,b show SEM images of BR-C cryogels prepared at *T*_cry_ = 18 and −2 °C, respectively. The disordered round-shape pores, formed at *T*_cry_ = −18 °C, become more ordered and aligned when *T*_cry_ is increased to −2 °C. A similar observation was reported before and attributed to the slow freezing of the reaction system at *T*_cry_ close to the freezing point of the reaction system [36]. Slow freezing of the reaction system provides growth of solvent crystals in the direction from surface to the interior of the reactor by creating an aligned porous structure in the cryogels. However, under fast freezing conditions, such as at *T*_cry_ = −18 °C, an ordered pore morphology will not form due to the formation of a larger number of solvent crystals growing in all directions. Due to the distinctly different morphologies of BR-C cryogels formed at −2 and −18 °C, we performed the mechanical tests on both BR-C cryogels to highlight the effect of the pore morphology on their mechanical properties.

### 2.4. Mechanical Properties and Self-Recoverability of Cryogels

**Figure 6:**
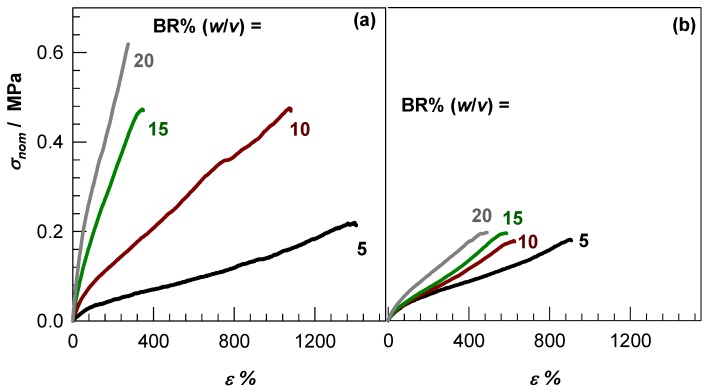
Tensile stress–strain curves of BR-C cryogels prepared at various BR% (*w*/*v*) and at *T*_cry_ = −2 °C (**a**) and −18 °C (**b**).

Stretchable cryogels with a high strength are required for many application areas, such as in oil spill removal from water. Although cryogels are tough materials under compression and can fully be compressed without any crack development [33], previous works show that they are brittle in tension. Figure 6a,b show tensile stress–strain curves of BR-C cryogels prepared at −2 and −18 °C, respectively, where the nominal stress *σ*_nom_ is plotted against the strain *ε*. Cryogels were prepared at various BR concentrations as indicated in the figures. It is seen that the cryogel prepared at 5% (*w*/*v*) BR and *T*_cry_ = −2 °C sustains up to around 1400% stretch ratios. To our knowledge, such a highly stretchable cryogel has not been reported before. The stretchability decreases as BR% (*w*/*v*) is increased, and becomes 300% at the highest BR concentration of 20% (*w*/*v*). Besides, cryogelation temperature *T*_cry_ also affects the flexibility of the cryogels. Stretchability, tensile strength, and the toughness, i.e., the area under the stress–strain curve, up to the fracture point, increase with increasing cryogelation temperature from −18 to −2 °C, revealing that formation of an aligned pore morphology contributes to the tensile mechanical properties of the cryogels.

**Figure 7:**
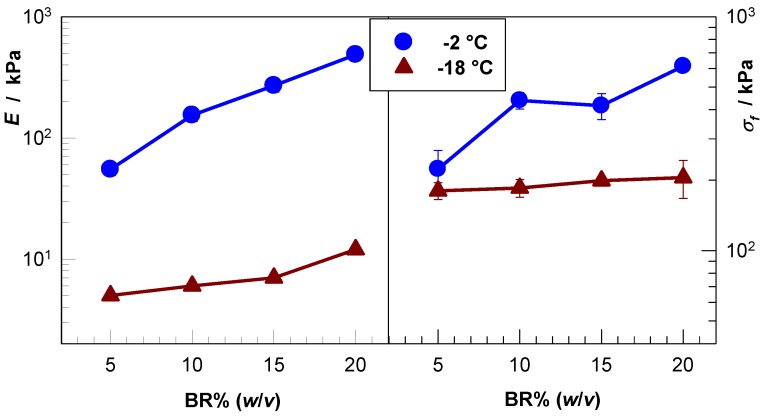
Young’s modulus *E* and fracture stress *σ_f_* of BR-C cryogels formed at −2 °C (circles) and −18 °C (triangles) plotted against BR% (*w*/*v*). *n* = 5, M ± SD. Error bars are smaller than the symbols where the bars are not shown.

Figure 7 shows the Young’s modulus *E* and fracture stress *σ_f_* of BR-C cryogels formed at −2 and −18 °C plotted against BR% (*w*/*v*). As expected, the modulus *E*, i.e., the crosslink density of the cryogels, increases with increasing BR% (*w*/*v*) because of the simultaneous increase of the number of elastically effective network chains per unit cryogel volume. However, this increase is much faster, and the modulus is much higher for cryogels formed −2 °C as compared to those formed at −18 °C. For instance, as BR concentration increases from 5% to 20% (*w*/*v*), *E* increases from 55 ± 6 to 490 ± 30 kPa at *T*_cry_ = −2 °C, whereas it slightly increases from 5 to 12 Pa at *T*_cry_ = −18 °C. The fracture stress *σ_f_* shows a similar trend and it increases with BR% (*w*/*v*), as well as with increasing cryogelation temperature. The highest fracture stress of 614 ± 17 kPa was observed at *T*_cry_ = −2 °C and 20% (*w*/*v*) BR. 

**Figure 8:**
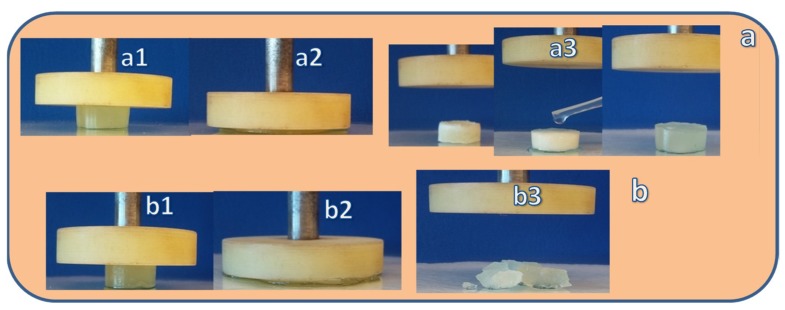
Photographs BR-C cryogels formed at −18 (**a**) and −2 °C (**b**) during the compression tests. BR = 15% (*w*/*v*). The images **a1**–**a3** show almost complete compression of a cryogel specimen formed at −18 °C under strain and immediate recovery of its initial shape after unloading. The images **b1**–**b3** show rupture of the cryogel specimen formed at −2 °C upon compression.

Uniaxial compression tests conducted on cryogels prepared at −18 °C revealed their almost complete and reversible compressibility. Although those formed at −2 °C were also completely compressible at a low BR concentration, a ductile-to-brittle transition was observed as the BR content is increased. For instance, the images in Figure 8a,b illustrate images of the cryogel specimens prepared at 15% (*w*/*v*) BR and at *T*_cry_ = −18 and −2 °C, respectively, during the compression tests. The specimen formed at −18 °C can be completely compressed under strain, during which toluene is released from the pores of the cryogel network (Figure 8a1–a2). After unloading, addition of toluene immediately recovers the initial shape of the gel specimen (Figure 8a3). However, the cryogel specimen synthesized at −2 °C breaks into several pieces upon compression, as is typical for classical hydrogels and organogels (Figure 8b1–b3). This brittle behavior is attributed to the formation of non-porous organogels at high BR contents, due to freezing point depression.

**Figure 9:**
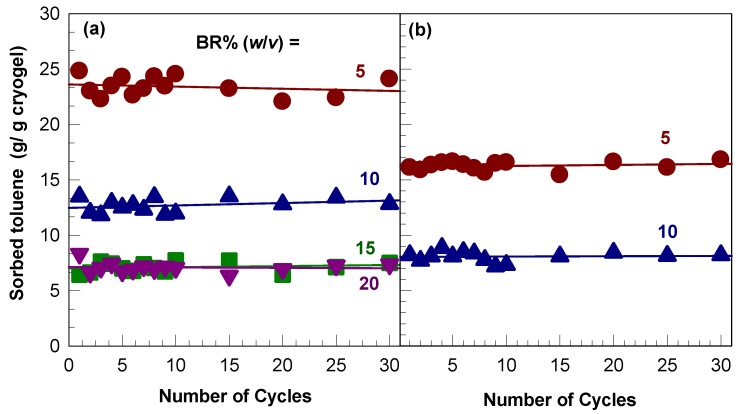
Sorption capacity of BR-C cryogels prepared at various BR% (*w*/*v*) indicated. *T*_cry_ = −18 °C (**a**) and −2 °C (**b**). The lines are best linear fits to the data. BR% (*w*/*v*) = 5 (●), 10 (▲), 15 (■), and 20 (▼).

The large and reversible compressibility of the cryogels provided their reusability as oil sorbents and, hence, the recoverability of the absorbed oil or organic solvents, such as toluene, after a simple squeezing step. Figure 9a,b demonstrate this behavior, where the amount of sorbed toluene in each sorption/squeezing cycle is plotted against the number of cycles for BR-C cryogels formed at −18 and −2 °C, respectively. For all cryogels, the amount of toluene sorbed in each cycle is independent of the number of cycles, at least up to 30 cycles, revealing that they can be used as reusable sorbents for organic pollutants, as well as for oil spill removal from water. For a given BR concentration, cryogels prepared at −18 °C are more effective as compared to those formed at −2 °C. For instance, at 5% (*w*/*v*) BR, the sorption capacities are 23.4 ± 0.9 and 16.3 ± 0.4 for *T*_cry_ = −18 and −2 °C, respectively. Increasing BR% (*w*/*v*) decreases the sorption capacity and at 10–15% (*w*/*v*) BR, the capacity reduces to 7.1 ± 0.5 for cryogels formed at −18 °C, whereas those formed at −2 °C become non-squeezable, like the classical sorbents. 

## 3. Conclusions

Cryotropic gelation processes have become more and more popular since the end of the 1980s because of the extraordinary properties of the resulting cryogels. Here, we presented the preparation and characterization of organo-cryogels based on BR under various experimental conditions. Benzene and cyclohexane were used as the solvents, whereas S_2_Cl_2_ was used as a crosslinker in the gel preparation. We have to mention that an ecofriendly solvent, instead of benzene or cyclohexane, would be a better alternative for the preparation of BR cryogels. However, our previous work shows that both benzene or cyclohexane are good solvents for BR with favorable freezing temperatures (~6 °C) for cryogelation reactions of BR [32]. It was found that the amount of the crosslinker, between 6% and 20%, has no effect on the swelling and mechanical properties, as well as on the morphology of the cryogels. However, the type of solvent, BR concentration, and the cryogelation temperature significantly affect the cryogel properties and morphologies. Benzene produces larger pore volumes as compared to cyclohexane due to the phase separation of BR from benzene at low temperatures, producing additional pores. Increasing cryogelation temperature from −18 to −2 °C leads to the formation of more ordered and aligned pores in the cryogels. Increasing BR content decreases the amount of unfrozen microphase of the frozen reaction solution, leading to a decrease in the total porosity and average diameter of the pores. A transition from cryogel to non-porous organogel was observed at high BR contents. Cryogels formed at −2 °C and at 5% (*w*/*v*) BR in cyclohexane sustain up to around 1400% stretch ratios. Cryogels swollen in toluene can completely be squeezed under strain, during which toluene is released from their pores, whereas addition of toluene to the squeezed cryogels recover their original shapes. BR cryogels presented here are suitable materials as reusable sorbents and passive samples for organic solvents, crude oil, and refinery products.

## 4. Materials and Methods

### 4.1. Materials

Butyl rubber (BK-1675N, Togliatti, Russia) containing 1.5 mol % isoprene units, sulfur monochloride (S_2_Cl_2_, Sigma Aldrich, St. Louis, MO, USA), cyclohexane (Merck, Kenilworth, NJ, USA), benzene (Merck), toluene (Tekkim, Istanbul, Turkey), and methanol (Tekkim) were used as received.

### 4.2. Preparation of the Cryogels

Butyl rubber (BR) cryogels were prepared in cyclohexane and in toluene at various BR and S_2_Cl_2_ concentrations at both −18 and −2 °C. Briefly, BR (5–20 g) was first dissolved in 100 mL of cyclohexane or benzene at room temperature overnight to obtain a homogeneous solution. After addition of different amounts of S_2_Cl_2_ and stirring for 2 min, the solution was transferred into several plastic syringes of 17 mm in diameter. Finally, syringes were immersed in a cryostat at −18 and −2 °C to conduct the cryogelation reactions. Synthesis conditions of the cryogels are summarized in Table 1.

### 4.3. Characterization of the Cryogels

Cryogels taken out of the syringes after a reaction time of 24 h were cut into small pieces of about 1 cm in length, and they were immersed in an excess of toluene at room temperature. After reaching an equilibrium degree of swelling, which was monitored by recording the mass and the diameter of the gel specimens, they were transferred into methanol, a poor solvent for BR. Finally, the specimens were dried under vacuum to constant mass. The gel fraction *W*_g_, i.e., the fraction of crosslinked rubber obtained from one gram of BR was calculated as

(2) $$W_{g}=\frac{m_{\mathrm{dry}}}{m_{0}\;w_{\mathrm{BR}}},$$

where *m*_dry_ and *m*_0_ are the weights of the gel specimens in dried and as-prepared states, respectively, and *w*_BR_ is the weight fraction of BR in the initial reaction solution. The equilibrium weight *q*_w_ and volume swelling ratios *q_v_* of the cryogels in toluene were calculated as

(3a) $$q_{w}=\frac{m_{s}}{m_{\mathrm{dry}}},$$

(3b) $$q_{v}={\left(\frac{D_{s}}{D_{\mathrm{dry}}}\right)}^{3},$$

where *D*_dry_ and *m*_dry_ are the diameter and mass of the specimens in dried state, respectively, and *D*_s_ and *m*_s_ are the same quantities, but of the equilibrium swollen specimens. In addition, relative weight (*m*_rel_) and volume (*V*_rel_) swelling ratios with respect to the as-prepared state were calculated as

(4a) $$m_{\mathrm{rel}}=\frac{m_{s}}{m_{0}},$$

(4b) $$V_{\mathrm{rel}}={\left(\frac{D_{s}}{D_{0}}\right)}^{3}.$$

To determine the pore volume *V*_p_ of the cryogels, dried cryogel specimens were immersed in an excess of methanol and their equilibrium mass *m*_M_ were recorded. The pore volume *V*_p_, that is, total volume of the pores in unit mass of dry BR network was estimated using the equation [36]

(5) $$V_{p}=\frac{m_{M}-m_{\mathrm{dry}}}{d_{M}\;m_{\mathrm{dry}}},$$

where *d*_M_ is the density of methanol (0.792 g/mL).

For texture determination, scanning electron microscopy (SEM, Jeol JSM 6335F Field Emission SEM) measurements were carried out on dried cryogels at various magnifications, and the average pore diameters *D* were calculated.

To determine the mass fraction *f* of unfrozen solvent in apparently frozen BR solutions at −18 °C, differential scanning calorimetry (DSC) measurements were carried out on a Perkin-Elmer Diamond DSC. For this purpose, about 30 mg of frozen cyclohexane solution containing various amounts of BR was placed in an aluminum sample pan of the instrument. After sealing and weighing the pan (*m*_1_), it was frozen within the instrument at −18 °C and then heated to 15 °C with a rate of 1 °C/min to determine the melting enthalpy *ΔH* of frozen cyclohexane. Finally, the pan was punctured and dried at 80 °C to constant weight (*m*_2_). The fraction *f* was calculated as

(6) $$f=1-\frac{\Delta H/\Delta H_{m}}{m_{\mathrm{chh}}},$$

where *ΔH*_m_ is the melting enthalpy (in J/g) of cyclohexane, and *m*_ch_ is the weight of cyclohexane in the sample pan, calculated as *m*_ch_ = *m*_1_ − *m*_2_.

Uniaxial tensile tests were performed at room temperature on a Zwick Roell mechanical test machine using 500 N load cell. For tensile tests, cryogel specimens were prepared in the form of sheets of about 2 mm thickness within the petri dishes. Tensile test data were collected at a strain rate of 50 mm/min. Young’s modulus *E* was calculated from the slope of stress–strain curves between 5% and 10% elongations. The stress was calculated as its nominal *σ*_nom_ value, which is the force per cross-sectional area of undeformed specimen, while the strain was given by the elongation ratio *ε*. Uniaxial compression tests were also conducted to demonstrate the self-recoverability and reusability of BR cryogels. Successive sorption–compression cycles were conducted on equilibrium swollen cryogel specimens in toluene. They were first compressed and then the mass of the squeezed samples was recorded. After addition of toluene, this step was repeated 30 times. Finally, cryogel specimens were dried to constant mass to calculate the sorbed amount of toluene by one gram of dry cryogel. Note that for each cryogel, at least five swelling, porosity, and mechanical measurements from different specimens were averaged.

## Figures and Tables

**Table 1:** Synthesis conditions of the cryogels.

Cryogel Code	Solvent	BR% (*w*/*v*)	S_2_Cl_2_% (*v*/*w*)	*T*_cry_ (°C)
BR-C	Cyclohexane	5–20	0–20	−2 and −18
BR-B	Benzene	5–20	0–20	−18

## Supplementary Materials

They are available online at http://www.mdpi.com/2310-2861/5/1/1/s1.
