# Supplementary material for: Highly Stretchable and Rapid Self-Recoverable Cryogels Based on Butyl Rubber as Reusable Sorbent
Source: Gels. 2019 Jan 7;5(1):1. doi: 10.3390/gels5010001 (PMC6473387; doi:10.3390/gels5010001)
Supplement: Supplementary file 1 [file gels-05-00001-s001.pdf]

Supplementary Information

Highly-Stretchable and Rapid Self-Recoverable Cryogels Based on Butyl Rubber

Sevil Muslumova , Berkant Yetiskin and Oguz Okay

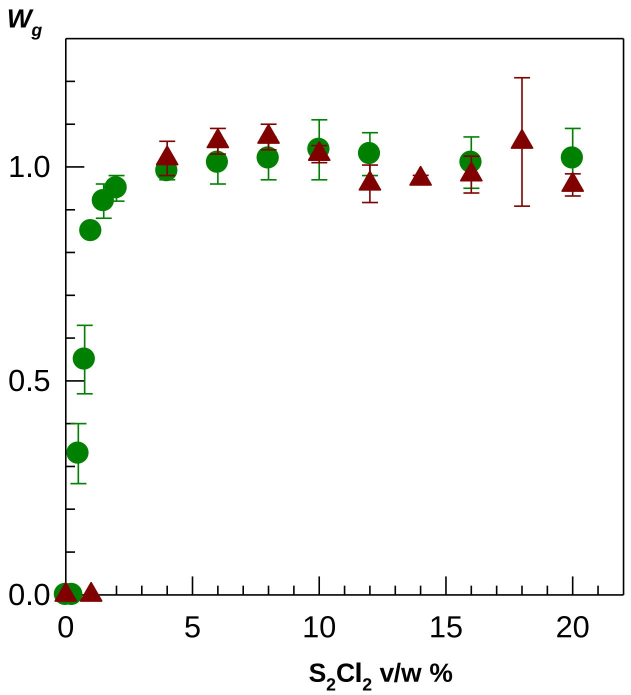

Figure S1. Gel fraction W_g of BR-B (circles) and BR-C (triangles) cryogels plotted against S_2Cl_2 concentration. BR = 5 w/v % and T_{cry} = - 18 °C.

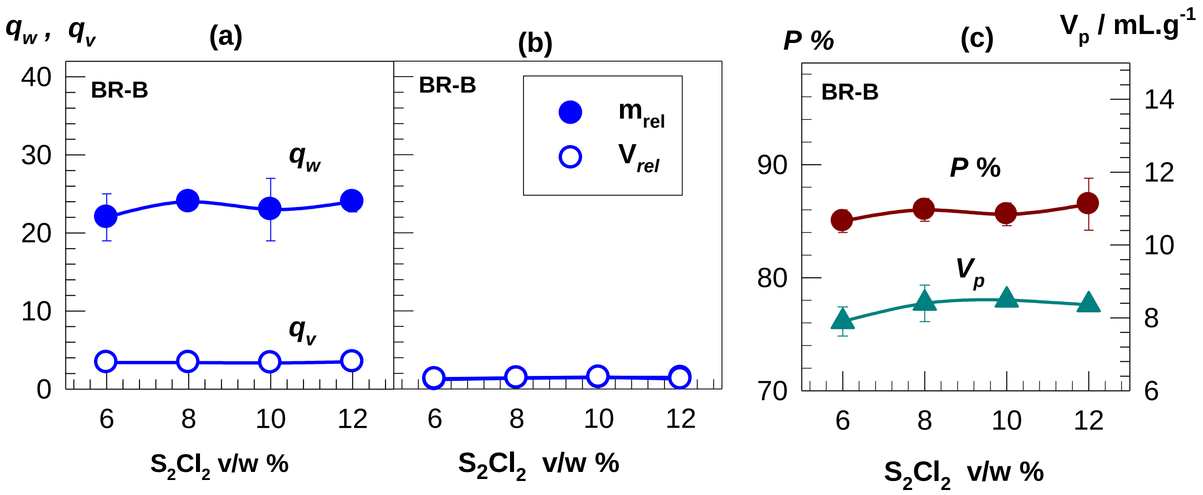

Figure S2. Weight and volume swelling ratios and porosities BR-B cryogels shown as a function of S_2Cl_2 concentrations. T_{cry} = -18 °C. BR = 5 w/v %.

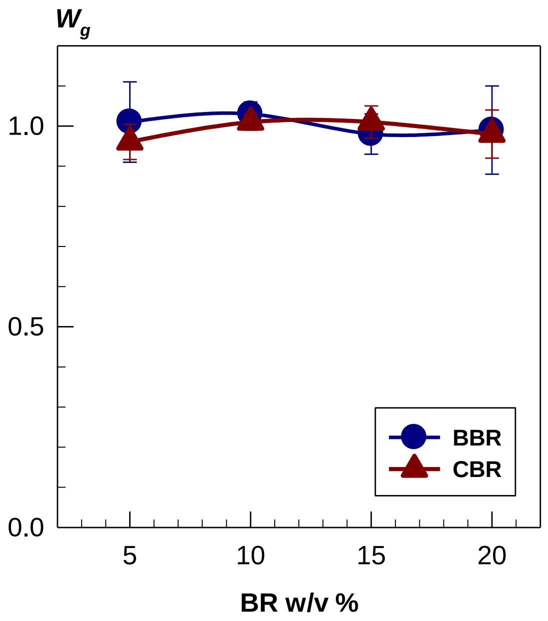

Figure S3. Gel fractions W_g of BR-B (circles) and BR-C cryogels (triangles) plotted against BR concentration. T_{cry} = -18 °C. S_2Cl_2 = 10 v/w %.

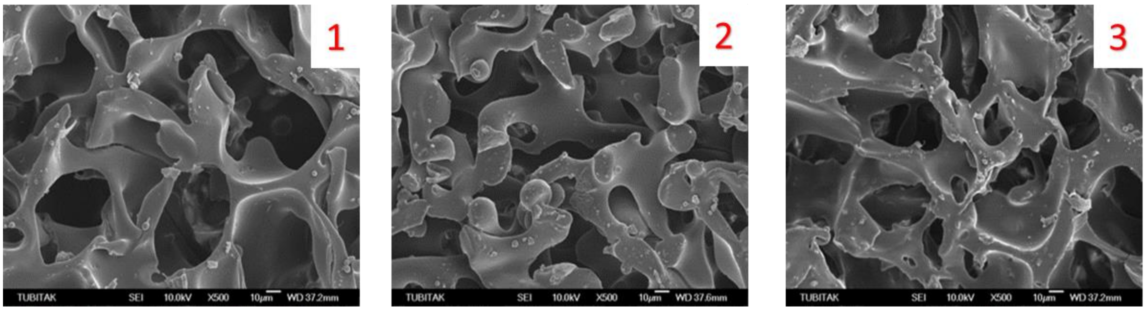

Figure S4. SEM images of BR-C synthesized at S_2Cl_2 = 12 (1), 18 (2) and 20 v/w % (3). BR = 5 w/v %. T_{cry} = -18 °C.
